# FINANCIAL EXPLOITATION IN OLDER AGE: A REVISIONARY MODEL

**DOI:** 10.1093/geroni/igad104.0881

**Published:** 2023-12-21

**Authors:** Duke Han

**Affiliations:** University of Southern California, Los Angeles, California, United States

## Abstract

Financial exploitation can have a devastating impact on the independence and wellbeing of older adults, yet the reasons why some older adults experience financial exploitation remain elusive. Recent work informed by the fields of neuropsychology, neuroscience, behavioral economics, and epidemiology has increasingly demonstrated links between financial vulnerability in older age and serious health outcomes such as cognitive decline and incident Alzheimer’s Disease. Because of this, research on financial exploitation in older age has progressively been made a public health priority, and this has led to a substantial expansion in knowledge on the topic. Despite this beneficial growth in understanding, gaps in knowledge, misconceptions, and ageist viewpoints continue to persist. To address these, this presentation will (1) discuss the potential role of age-associated cognitive changes in financial exploitation, (2) review neuroimaging findings from our group and others relevant to understanding possible brain changes involved, and (3) highlight cross-cultural, interpersonal, and other considerations that need prioritization in future research endeavors. In conclusion, we will present a revisionary model of financial exploitation in older age which considers not only the potential vulnerabilities of the older adult, but also the persons committing the financial exploitation and the contextual factors that may contribute to poorer outcomes. This revisionary model can more effectively inform interventions for the prevention and mitigation of financial exploitation in older age.

